# Pathological Neurovascular Unit Mapping onto Multimodal Imaging in Diabetic Macular Edema

**DOI:** 10.3390/medicina59050896

**Published:** 2023-05-07

**Authors:** Tomoaki Murakami, Kenji Ishihara, Noriko Terada, Keiichi Nishikawa, Kentaro Kawai, Akitaka Tsujikawa

**Affiliations:** Department of Ophthalmology and Visual Sciences, Kyoto University Graduate School of Medicine, Kyoto 606-8507, Japan

**Keywords:** center-involving diabetic macular edema, clinically significant macular edema, diabetic macular edema, diabetic retinopathy, disorganization of retinal inner layers, fluorescein angiography, fluorescein leakage, fundus autofluorescence, hard exudates, hyperreflective foci, optical coherence tomography, optical coherence tomography angiography, photoreceptor damage, vascular hyperpermeability

## Abstract

Diabetic retinopathy is a form of diabetic microangiopathy, and vascular hyperpermeability in the macula leads to retinal thickening and concomitant reduction of visual acuity in diabetic macular edema (DME). In this review, we discuss multimodal fundus imaging, comparing the pathogenesis and interventions. Clinicians diagnose DME using two major criteria, clinically significant macular edema by fundus examination and center-involving diabetic macular edema using optical coherence tomography (OCT), to determine the appropriate treatment. In addition to fundus photography, fluorescein angiography (FA) is a classical modality to evaluate morphological and functional changes in retinal capillaries, e.g., microaneurysms, capillary nonperfusion, and fluorescein leakage. Recently, optical coherence tomography angiography (OCTA) has allowed us to evaluate the three-dimensional structure of the retinal vasculature and newly demonstrated that lamellar capillary nonperfusion in the deep layer is associated with retinal edema. The clinical application of OCT has accelerated our understanding of various neuronal damages in DME. Retinal thickness measured by OCT enables us to quantitatively assess therapeutic effects. Sectional OCT images depict the deformation of neural tissues, e.g., cystoid macular edema, serous retinal detachment, and sponge-like retinal swelling. The disorganization of retinal inner layers (DRIL) and foveal photoreceptor damage, biomarkers of neurodegeneration, are associated with visual impairment. Fundus autofluorescence derives from the retinal pigment epithelium (RPE) and its qualitative and quantitative changes suggest that the RPE damage contributes to the neuronal changes in DME. These clinical findings on multimodal imaging help to elucidate the pathology in the neurovascular units and lead to the next generation of clinical and translational research in DME.

## 1. Introduction

Diabetic retinopathy (DR) is one of the leading causes of vision loss worldwide. In particular, diabetic macular edema (DME) and proliferative diabetic retinopathy (PDR) are vision-threatening DR [1]. Although the standard clinical practice for DME has been established, the pathogenesis of DME remains to be fully characterized and further advances in diagnosis and treatment should be pursued [2,3,4].

DME is characterized by vascular hyperpermeability and retinal edema in the macula, resulting in reduction of visual acuity (VA) [4]. Classical fundus examination has allowed us to evaluate and diagnose DME [2]. The distinct findings are retinal hemorrhages, hard exudates, and microaneurysms. The thickening of translucent retinas is the most relevant, although its subjective evaluation is difficult even for the experienced clinicians.

Histological publications have elucidated the morphological changes and their components in DME [5,6,7,8,9,10]. Cystoid macular edema (CME) often develops in the inner nuclear layer (INL) and outer plexiform layer (OPL) in eyes with macular edema [10]. Electron microscopy suggested that these spaces correspond to the accumulation of the extracellular fluid or intracytoplasmic swelling due to the liquefaction necrosis of neuroglial cells [11]. Tight junctions are abundantly developed between vascular endothelial cells in healthy retinas, whereas they are disrupted in diabetic retinas [12,13,14]. Trypsin-digested specimens revealed that pericyte loss and vascular deformation, i.e., microaneurysms, may contribute to vascular hyperpermeability [5,15]. Hard exudates may correspond to the accumulation of lipid-laden macrophages or the deposition of lipoproteins in the histological samples [16,17]. These publications allow clinicians to speculate about the pathogenesis of DME.

Advances in vascular biology have elucidated that vascular endothelial growth factor (VEGF) plays the most important role in angiogenesis and vascular hyperpermeability in PDR and DME, respectively [18,19,20]. This prompted us to apply anti-VEGF treatment for DME [21,22,23]. Among several therapeutic strategies, it is the first-line therapy and regulates vascular hyperpermeability most efficiently [24]. Currently, clinicians are trying to determine the predictors of visual outcomes, treatment frequency, and remission of macular edema after anti-VEGF treatment to pursue customized medicine [25,26,27,28,29,30].

**Figure 1:**
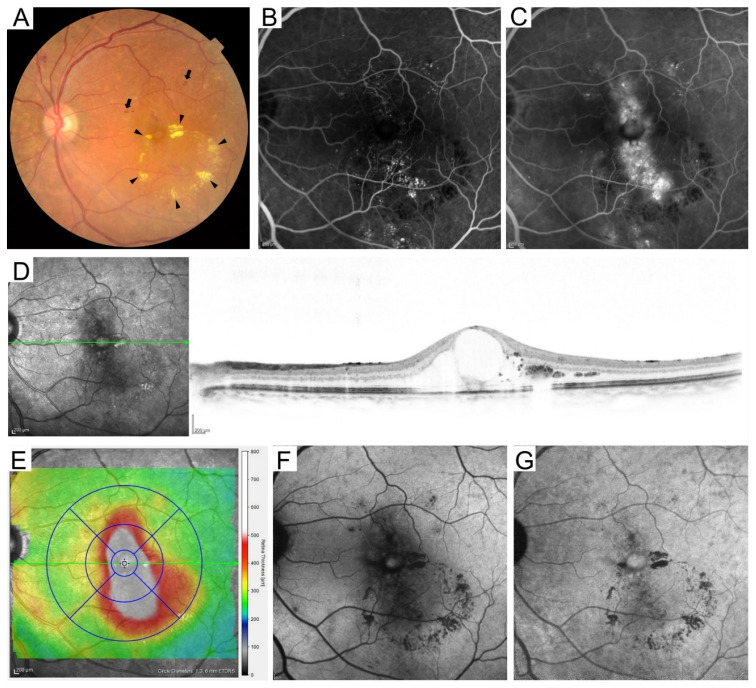
Multimodal imaging in a representative case with diabetic macular edema. (**A**) Retinal hemorrhages (arrows) and hard exudates (arrowheads) in fundus photography. The early (**B**) and late (**C**) phases of FA images show fluorescein leakage and pooling. (**D**) Cystoid macular edema is delineated on the sectional optical coherence tomography (OCT) image obtained using Spectralis OCT along the green arrow on the scanning laser ophthalmoscope image. (**E**) Two-dimensional OCT thickness map (total retinal thickness) with an ETDRS grid overlay (1, 3, 6 mm-circles). The central subfield thickness is 651 μm. Different OCT machines use different segmentation methods for the outer boundaries of retinas, so the mean retinal thicknesses depend on the OCT machines. (**F**) Short wavelength fundus autofluorescence. (**G**) Near-infrared FAF.

In this article, we searched PubMed as an electronic database, and selected the first publication in the relevant field. We discuss about how the recent application of multimodal imaging improves our understanding of the pathogenesis of DME and resolves the clinical issues, e.g., visual prognosis (Figure 1).

## 2. Clinical Diagnosis of DME

At present, many clinicians objectively and quantitatively diagnose center-involving diabetic macular edema (CIDME) using optical coherence tomography (OCT) [3]. It is reasonable to designate the retinal thickening in the macula as the diagnostic criterion for DME. CIDME is used as the eligibility criterion in most clinical trials of anti-VEGF treatment [31]. Another classical diagnosis of DME is clinically significant macular edema (CSME) [2]. The criteria are retinal thickening or hard exudates within 500 μm from the fovea or 1500 μm or more of retinal thickening within 1 disc diameter of the fovea on fundus examination or the stereoscopic fundus photography. The Early Treatment Diabetic Retinopathy Study (ETDRS) had defined CSME as the criterion for macular photocoagulation in DME.

In addition to these two major diagnostic criteria, the international classification of diabetic macular edema disease severity depends on the proximity of the retinal edema and hard exudates to the fovea [32]. Eyes with retinal thickening or hard exudates in the posterior pole were diagnosed as DME. Eyes with these lesions distant from the center are defined as mild DME. When these lesions are approaching or involving the center, we diagnose moderate or severe DME, respectively. Finally, fluorescein angiography (FA) shows focal and/or diffuse fluorescein leakage as a biomarker of vascular hyperpermeability [33].

The pathogenesis of DME is complicated, and evidence is continuing to accumulate using newly developed imaging modalities, so we have not reached a consensus on the ultimate criteria for DME diagnosis.

## 3. Fundus Photography

### 3.1. Conventional Photography

**Figure 2:**
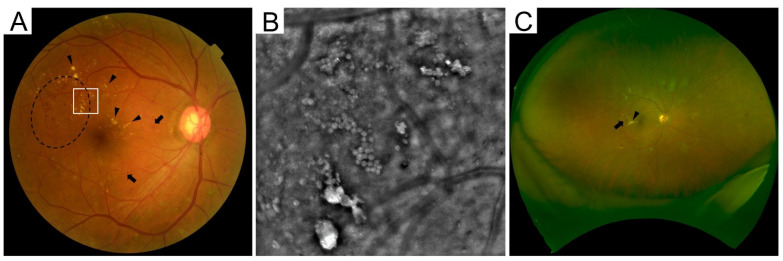
Various instruments for fundus photography. (**A**) Clinically significant macular edema on conventional photography. Arrows and arrowheads indicate retinal hemorrhages and hard exudates, respectively. Dashed line is retinal thickening. (**B**) The high-resolution image by adaptive optics camera within the square in panel A reveals that hard exudates are composed of multiple round lesions. (**C**) Ultrawide-field scanning laser ophthalmoscope images can show approximately 80% of the area of the entire retina.

Fundus examination was originally the basis of the diagnosis and treatment of DME (Figure 2A). Fundus photography is useful for longitudinal evaluation. Their main findings are retinal hemorrhages, hard exudates, and retinal thickening in the macula, all of which represent the breakdown of the blood-retinal barrier (BRB) [4]. In particular, VA is reduced when the foveal center is involved with retinal edema and hard exudates. The ETDRS defined CSME depending on the fundus findings and demonstrated that VA reduction is retarded by macular photocoagulation [2].

Hard exudates are generally considered to be the accumulation of the extravasated lipoproteins or the lipid-laden macrophages [16,34]. They are sometimes arrayed at the edge of retinal edema, which is referred to as circinate hard exudates. This suggests the vascular lesions with hyperpermeability at the center of the “circinate”. When they migrate into the subfoveal spaces, they often lead to the photoreceptor damage at the fovea and subsequent VA reduction [35,36].

### 3.2. Advanced Technologies

There are three main advances in fundus photography: ultrawide field imaging, super high resolution, and separate wavelengths. The clinical application of ultrawide-field fundus photographs has significantly improved the quality of the assessment of peripheral retinas (Figure 2B) [37]. However, we carefully evaluate the macular lesions, because the lateral resolution is slightly reduced in these indirect images. Adaptive optics technology, which was originally applied to astrology to remove the wavefront aberrations, has increased the lateral resolution of fundus cameras and scanning laser ophthalmoscopes (SLOs) [38,39]. As a result, we can appreciate the fine structure of vascular lesions, hard exudates, and photoreceptor mosaics (Figure 2C). Since the SLO obtains the fundus imaging, mediated via specific wavelengths of emission and excitation, the light reflection in the SLO images delineates the unusual fundus images. The clinical feasibility of such modalities should be elucidated.

## 4. Dye-Based Angiography

### 4.1. Fluorescein Angiography

**Figure 3:**
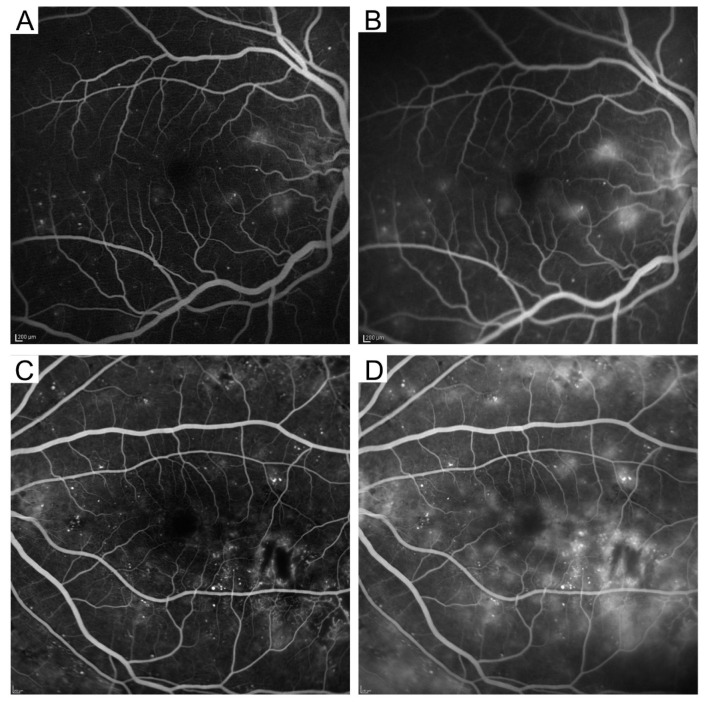
Two major patterns of fluorescein leakage on fluorescein angiography images. Focal (**A**,**B**) and diffuse (**C**,**D**) fluorescein leakage. The early (**A**,**C**) and late (**B**,**D**) phases.

FA is the best imaging modality to assess the vascular morphologies and dysfunction in the retinas. Higher contrast allows us to appreciate capillaries and details of the morphological changes in retinal vessels. In particular, FA is the only modality to evaluate the BRB status. Fluorescein dye is extravasated into the retinal parenchyma in cases of BRB disruption, which is referred to as fluorescein leakage. It is classified into two patterns: focal and diffuse fluorescein leakage (Figure 3) [33]. When the source of the leaked dye can be identified clearly, the extravascular hyperfluorescence is considered focal fluorescein leakage. In most cases, microaneurysms are the source of dye leakage. In contrast, when the source is not distinctive, hyperfluorescence is referred to as diffuse fluorescein leakage. Focal and diffuse leakages often coexist, and they are not definitively divided. Another sign of the BRB breakdown is fluorescein pooling. Fluorescein dye is stored in cystoid spaces in the INL or OPL and appears to be round or oval.

FA has higher sensitivity to detect microaneurysms than fundus examination. Microaneurysms appear to be hyperfluorescent dots and are sometimes accompanied by fluorescein leakage [5,15]. FA is useful to discriminate microaneurysms from dot-like retinal hemorrhages, which correspond to blocked fluorescence. Circinate hard exudates in fundus examination and microaneurysms with leakage often coexist. When we consider macular photocoagulation for CSME, FA images are useful to determine whether vascular lesions are coagulated.

The disadvantages of FA also need to be considered. The retinal vasculature is composed of three or four capillary plexus layers. However, the signals on two-dimensional images are derived mainly from the superficial layer, and we cannot assess the status of deep vascular plexuses on FA images [40]. Another consideration is patient allergy, for which first aid for anaphylaxis needs to be prepared.

### 4.2. Indocyanine Green Angiography

Indocyanine green angiography (ICGA) is another imaging modality of chorioretinal vessels. This dye has an affinity for lipoproteins, and some microaneurysms are delineated as hyperfluorescent dots on ICGA images. It was reported that such microaneurysms are the source of vascular leakage and might be appropriate targets for focal macular photocoagulation [41].

## 5. Optical Coherence Tomography

Clinical application of spectral-domain optical coherence tomography (SD-OCT) has deepened our understanding of DME in neuroglial tissue [42,43]. One of the main characteristics of DME is retinal thickening. SD-OCT allows us to evaluate retinal edema qualitatively and quantitatively [44]. In particular, the mean retinal thickness in the central subfield, referred to as central subfield thickness (CST), is used to diagnose CIDME [3,31]. Some lesions develop, and some important structure in the retinas is lost on SD-OCT images in eyes with DME.

### 5.1. Principles

OCT is a noninvasive imaging modality. Time-domain (TD)-OCT, the original technique, depends on the coherence between the reflected lights from the retinas and the reference mirror [45]. As a result, we can differentiate the retinal layers according to OCT reflectivity. In SD-OCT with approximate 840 nm light source, the nonuniform Fourier transform of OCT signal spectra increased the scan speed and generated the high-resolution images (axial resolution = 3–5 μm) [42]. The latest generation is swept source (SS)-OCT [46]. The longer wavelength of the light source (approximate 1040–1060 nm) penetrates deep structures, e.g., the choroid and sclera, in most of commercially available SS-OCT machines, and the axial resolution is considered to be 6–8 μm. As a result, SS-OCT enables us to evaluate the chorioretinal structure. Visible light is sometimes used as the light source for the research. Of these, SD-OCT is the main technique used today. We need to pay attention to the several artifacts including motion artifacts, blinking artifacts, and segmentation error. Hyperreflective lesions often lead to the shadow beneath themselves.

In the research field, adaptive optics technologies have been introduced into the OCT imaging [47]. These technologies have improved lateral resolution, and some structures, e.g., ganglion cells and photoreceptor cells, may be delineated in a single-cell manner [48].

### 5.2. Diagnosis of CIDME

**Figure 4:**
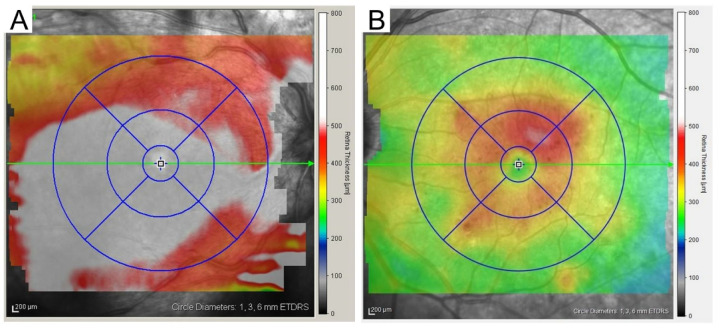
Two-dimensional optical coherence tomography (OCT) map. A two-dimensional map with an ETDRS grid overlay (1, 3, 6 mm-circles), which is obtained using Spectralis OCT, allows us to discriminate center-involving diabetic macular edema (CIDME) (**A**) from non-CIDME (**B**).

Retinal sectional images are obtained using OCT, and the side-by-side arrangement of these images constructs three-dimensional images, which allows us to measure the retinal thickness in each sector of the ETDRS grid. In particular, the mean retinal thickness within the central 1-mm circle is defined as the CST. When the CST is greater than the thresholds, eyes with DR are diagnosed with CIDME (Figure 4, Table 1) [3]. We must be careful with this quantitative diagnosis. The thresholds are different in the different devices, because of the different methods used for automatic segmentation [31]. In some cases, the segmentation is incorrect, and as a result, the measurement is also incorrect. We may consider the manual measurement using the caliper in the equipped software.

This quantified parameter is often applied as a surrogate marker in clinical trials for DME. We can confirm improvement of the edematous changes and remission of CIDME using the CST [29,49]. Although the CST is the gold standard for the structural assessment of DME, its association with VA reduction is modest. We sometimes observe the cases with paradoxical VA changes, e.g., VA reduction in eyes with decreased CST and VA improvement in eyes with increased CST [50]. This suggests that factors other than retinal edema influence visual function [4,51,52].

### 5.3. Various Pathomorphologies on Sectional Images

**Figure 5:**
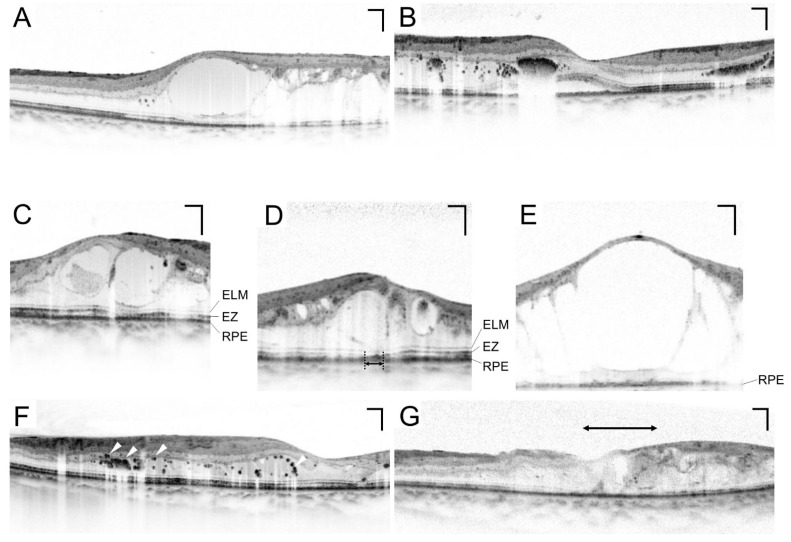
Various findings on sectional optical coherence tomography (OCT) images. The images obtained using Spectralis OCT show pathomorphologies, e.g., cystoid macular edema (**A**) and serous retinal detachment (**B**). Intact (**C**), disrupted (**D**) (double-headed arrow), and absent (**E**) ellipsoid zone (EZ) lines. ELM = external limiting membrane; RPE = retinal pigment epithelium. (**F**) Hyperreflective foci (white arrowheads). (**G**) DRIL as a biomarker of neural damage (double-headed arrow). Scale bar = 200 μm.

OCT delineates various lesions in neuroglial tissues in DME. The first publication of the pathomorphologies in DME documented three patterns on TD-OCT images: CME, serous retinal detachment (SRD), and sponge-like retinal swelling (Figure 5A,B) [53]. They considered that retinal thickening is composed of one or a mixture of these structural lesions. High-resolution SD-OCT images have shown that CME develops mainly in the INL and OPL and that sponge-like swelling results from epiretinal membrane or taut posterior hyaloidal membrane [54,55].

Comparative studies between FA and OCT images have promoted our understanding of the multifaceted pathogenesis in DME. Honeycomb and petaloid patterns of fluorescein pooling correspond to cystoid spaces in the INL and OPL, respectively [54]. The foveal avascular zone (FAZ) is enlarged, and microaneurysms around it develop in CME eyes [56]. This suggests that retinal ischemia and microaneurysms contribute to the development of CME. In contrast, we rarely find the fluorescein pooling in SRD eyes. Perifoveal hyperfluorescence is often observed in SRD eyes, so we hypothesize that the distant effects from vascular hyperpermeability in the perifovea result in the development of SRD [57]. This correlation may prompt us to perform the customized methods of macular photocoagulation.

### 5.4. Photoreceptor Damage

High-resolution images in the outer retinal layers allow us to appreciate the pathological changes in the photoreceptor-retinal pigment epithelium (RPE) complex. SD-OCT delineates the ellipsoid zone (EZ) and the external limiting membrane (ELM) above the RPE [58], which are biomarkers of the photoreceptor status. In some cases with DME, one or both of these lines are disrupted or absent at the fovea (Figure 5A–C) [59,60,61,62,63]. Since VA depends on the foveal photoreceptors in human eyes, their damage leads to VA reduction. The pathological mechanisms underlying photoreceptor damage remain to be elucidated. A recent publication has shown that anti-fumarase antibody is increased in some patients with DME and promotes the photoreceptor inner and outer segments in mice [64]. This suggests the autoimmune mechanisms in the photoreceptor damage in DME.

The absence or disruption of the EZ line is a biomarker of poor prognosis after some interventions for DME. Intriguingly, the status of EZ lines is partially restored after anti-VEGF treatment for DME [65,66].

### 5.5. Hyperreflective Foci

Hyperreflective foci have been reported as a sign of extravasation in DME [43]. Morphologically, dot-like deposits with high OCT reflectivity are delineated individually or aggregated (Figure 5F). Hard exudates in fundus examination correspond to the accumulation of hyperreflective foci. Surgically resected hard exudates contain phagocytes, and histological publications have showed lipid-laden macrophages in diabetic retinas [16]. Future clinicopathological study should elucidate the relationship between this OCT finding and histological lipid-laden macrophages [17,34].

Hyperreflective foci are delineated throughout the retina and often accumulate in the OPL. They are present in cystoid spaces or vascular walls. In some cases with DME, hyperreflective foci migrate into the subfoveal spaces and promote photoreceptor damage there [67,68]. Further studies should show how hyperreflective foci exacerbate neuroinflammation in DME [69].

### 5.6. Lamellar Disorganization

In healthy retinas, the signals from foveal photoreceptors are transmitted through inner retinal layers, including bipolar cells and ganglion cells. They are represented by the definite lamellar structures in inner retinas. In some eyes with DME, some or all inner layers are absent or disorganized, which is referred to as disorganization of the retinal inner layers (DRIL) (Figure 5G) [70]. The magnitude of DRIL is associated with VA reduction cross-sectionally and longitudinally.

Diabetic macular ischemia also induces VA reduction and is associated with DME, mediated via VEGF and insufficient drainage of extracellular fluids [71,72,73]. The contrast between the highly reflective nerve fiber layer (NFL) and the less reflective ganglion cell layer/inner plexiform layer (GCL/IPL) enables us to assess the integrity of the inner retinal layers. The boundaries between the layers are often absent or obscure in areas with capillary nonperfusion [74]. Such lesions might result in VA reduction collaboratively with DME.

### 5.7. Vitreoretinal Interface

DME is sometimes accompanied by epiretinal membrane or vitreomacular traction. Assessing these lesions is difficult even for the experienced clinicians. In contrast, OCT delineates them very clearly, which allows us to infer the magnitude of traction and subsequent visual dysfunction. Therefore, it is easy to determine whether vitrectomy should be performed in DME with vitreoretinal pathology [75,76].

### 5.8. Choroid

Many clinicians believe that the choroid influences on the pathogenesis of DME, because the choroid nourishes the outer retinas through the RPE and drains waste from the retinas [77]. Some publications reported greater choroidal thickness in eyes with DME, and others reported opposite results [78,79,80,81,82]. Ocular inflammation or vascular hyperpermeability might increase choroidal thickness, whereas the loss of choroidal vessels might decrease it. Choroidal thickness depends on axial length, age, and systemic factors, and future studies should elucidate the cause-and-effect relationship between DME and choroidal thickness [83,84,85].

En face images of SS-OCT or enhanced depth imaging (EDI)-OCT depict the choroidal vessels with an intermediate or large diameter in the Sattler’s and Haller’s layers, respectively. The subjective evaluation of these layers indeed showed reduced vascular density or tortuosity of choroidal vessels in DR [86,87,88]. These lesions correspond to the histological findings at least in part [89,90]. Despite the definite choroidal pathology in diabetic eyes, it remains to be elucidated whether such lesions promote ocular inflammation or reduce the drainage of extracellular fluids from the retinas [91]. In addition, studies should focus on the relationship between immune cell infiltration and hyperreflective foci in the choroid [92].

## 6. Optical Coherence Tomography Angiography

**Figure 6:**
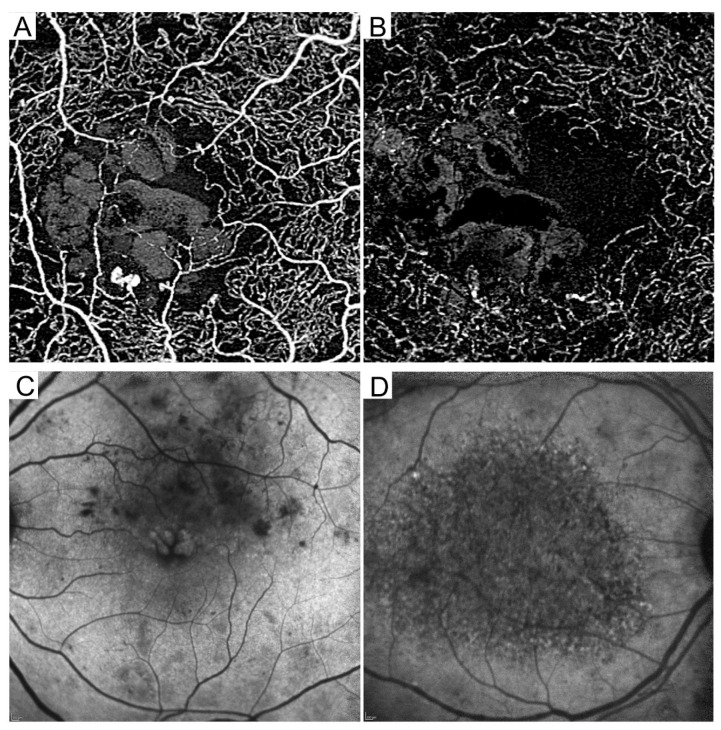
Latest modalities, optical coherence tomography angiography (OCTA) and fundus autofluorescence (FAF). Reduced vascular density in the superficial (**A**) and deep (**B**) en-face OCTA images. Scale bar = 200 μm. (**C**) Oval-shaped hyperfluorescence in short wavelength FAF. (**D**) Reduced levels of fluorescent signals in near-infrared FAF.

Optical coherence tomography angiography (OCTA) delineates three-dimensional retinal vasculature, depending on the differences in the optical reflectivity between sequential multiple B-scan images [93,94,95]. OCTA is the first device that can depict the deep capillary plexuses (Figure 6A,B) [96]. OCTA cannot be used to evaluate BRB breakdown. OCTA is in the process of clinical investigation and can demonstrate the pathogenesis of DME, although there is no consensus regarding the diagnostic criteria. We need to be careful of several artifacts in OCTA imaging [97,98]. In particular, motion artifacts often influence the image processing in diabetic patients with poor fixation. The main findings are microaneurysms, lamellar nonperfusion, and suspended scattering particles in motion (SSPiM) in cystoid spaces.

### 6.1. Microaneurysms

OCTA visualizes multiple morphologies of microaneurysms, e.g., fusiform and saccular, which may be very similar to the histological findings [5,99]. In contrast, microaneurysms present a dot-like appearance in fundus photography and FA images. Microaneurysms are delineated in the deep OCTA slab images, which is consistent with histological publications showing that many microaneurysms develop in the INL [100,101]. Several publications have focused on the association between vascular hyperpermeability and the characteristics of microaneurysms on OCTA images [102].

### 6.2. Lamellar Nonperfusion

Automatic quantification of vascular parameters can be applied on en face OCTA images because they have higher contrast and higher signal/noise ratios. The development of layer-by-layer vascular parameters are in progress, e.g., the size and morphological parameters of the FAZ, perfusion metrics (vascular density, vascular length density, and fractal dimension), and nonperfusion metrics (intercapillary area and total avascular area). In particular, retinal thickening or cystoid spaces are often accompanied by capillary nonperfusion in the deep layer [71,73]. It was reported that deep nonperfusion areas are related to photoreceptor damage [103]. An enlarged FAZ and reduced vascular density are predictors of poor visual outcomes after anti-VEGF treatment for DME [104,105]. Future studies should elucidate the relationship between diabetic macular ischemia and DME.

The OCTA slab images in the choriocapillaris layer present the mosaic-like appearance of flow signals in healthy eyes [77]. Loss of such OCTA signal is referred to as flow void in chorioretinal diseases. The flow void gradually increases according to the severity of DR [88]. Future studies should elucidate the pathological interactions between outer retinas and the RPE-choriocapillaris complex.

## 7. Fundus Autofluorescence

The RPE is the main component of the outer BRB and maintains metabolism in photoreceptor cells [106]. Fundus autofluorescence (FAF) enables us to evaluate the status of the RPE [107]. Two main modalities, short-wavelength (SW) FAF and near-infrared (NIR) FAF, have been clinically introduced. The SW-FAF and NIR-FAF signals are derived from the lipofuscin and melanin, respectively. Since the signal is faint, the media opacity reduces the signal/noise ratio.

The fluorescent signals on SW-FAF images gradually decrease to the fovea in healthy eyes because the signals from the RPE are blocked by the macular pigments in the retinal parenchyma. The signals are reduced in diabetic retinal pigment epitheliopathy. There are associations between FAF signals and visual functions in DME [108,109]. Oval-shaped hyperfluorescence corresponds to the foveal cystoid spaces in CME eyes (Figure 6C) [110].

The NIR-FAF signals increase to the fovea in healthy eyes, according to the density of melanin. A mosaic pattern on NIR-FAF images is often observed in eyes with DME and is associated with foveal photoreceptor damage and subsequent VA reduction [111,112]. Quantitative analysis revealed that NIR-FAF signal levels are negatively related to the logarithm of the minimum angle of resolution (logMAR) and CST (Figure 6D). It remains to be elucidated whether the RPE damage contributes to the exacerbation of DME or vice versa.

## 8. Emerging Questions

The advances in the modern modalities deepen our understanding the clinical findings on the classical modalities (Table 1). We have discussed the hot topics in fundus imaging rather than its systematic review. Resultantly, there might be bias, although its advances are raising interesting questions regarding the next generation of diagnosis and treatment for DME.

### 8.1. What Is DME?

We have discussed the clinical feasibility of each imaging modality above. Although there is no doubt that vascular hyperpermeability promotes DME, other mechanisms also contribute to its pathogenesis [4,51,52]. Retinal ischemia increases VEGF expression and subsequent breakdown of the BRB [113]. Capillary nonperfusion in the deep vascular plexus may reduce the ability to drain the extracellular fluids and concomitantly promote their storage [71,73]. Neurodegeneration may be represented by the DRIL and photoreceptor damage on OCT images and RPE changes on FAF images [59,60,70,109,111]. Hyperreflective foci and increases in cytokine levels may represent neuroinflammation in concert with vascular hyperpermeability [43,114]. These results suggest the necessity of an integrative understanding of DME pathology.

### 8.2. Will the Deep Learning Reduce Our Burden?

Artificial intelligence has been implemented in society and industry. In particular, deep learning is being applied to the medical issues, such as diagnosis, assistance during surgery, and drug discovery. The main concept of the deep learning is the convolutional neural network, which transforms from the complex information of several medical images to an all-or-nothing diagnostic decision. Gulshan and associates demonstrated, for the first time, the performance of the deep learning using fundus photography in the diagnosis of DR [115]. The study was followed by the publications that applied deep learning to OCT images for the automatic diagnosis of DME [116]. These technologies may take the place of the beginning ophthalmologists, or they may learn from the data from artificial intelligence in the medical diagnosis of vision-threatening DR.

Deep learning has an advantage in substituting the subjective evaluation, e.g., DR and CSME diagnosis using fundus photography [2,32]. In contrast, CIDME is objectively and quantitatively diagnosed using OCT, so the introduction of artificial intelligence is controversial [3]. We may apply deep learning to the assessment of subjective findings on sectional OCT images.

Despite its feasibility, we have to grasp the limitations of deep learning. It needs a training dataset, the quality of which influences the accuracy in the outcomes. The processes are in the black box, and these issues may reduce the reproducibility.

### 8.3. Can Fundus Imaging Offer Customized Medicine?

These imaging modalities deepen our understanding of the pathogenesis of DME. In particular, quantitative parameters, e.g., CST for reversible disease activity and DRIL and nonperfusion metrics for irreversible disease progression, are feasible in the longitudinal study. There are two major strategies to pursue optimized interventions. One strategy is that clinicians may plan therapeutic approaches against the pathogenesis specific to each patient. The other strategy is tailor-made medicine using prognostic factors.

The CST is the surrogate marker in the treatment of DME, although VA improvement and ME resolution are not necessarily consistent [50]. The multiple mechanisms discussed above might explain the paradoxical VA changes. Most circinate hard exudates are accompanied by leaking microaneurysms in their own center and can be treated by focal macular photocoagulation in eyes with CSME but not CIDME. Typical focal fluorescein leakage mostly derives from microaneurysms, which can be coagulated by LASER [33]. In contrast, diffuse fluorescein leakage should be treated by medical interventions including anti-VEGF treatment and ocular steroids [22,117]. Among the potential OCT findings, epiretinal membrane and vitreomacular traction are good biomarkers for vitrectomy even in the era of anti-VEGF therapy [75,118]. We may confirm photoreceptor restoration under anti-VEGF treatment, which is related to VA improvement [65]. This suggests that foveal photoreceptor status is a candidate surrogate marker for anti-VEGF treatment [66].

Some findings on fundus imaging modalities are reported as prognostic factors after intervention in DME. Classically, hard exudates at the fovea are a predictor of subretinal fibrosis and concomitant poor vision after macular photocoagulation for CSME [35,36]. Atrophic creep involving the fovea after photocoagulation also explains the significant reduction in VA. In the era of expensive anti-VEGF treatment, we must take both visual prognosis and socioeconomic burden into consideration. The presence of subretinal fluid is a predictor of visual gain after ranibizumab for DME [25]. The CST is positively associated with the treatment frequency of anti-VEGF drugs [26,27]. We can compare the prognostic factors between individual interventions for the optimized application of treatments.

## 9. Conclusions and Future Prospects

Multimodal fundus imaging sheds light on the clinical aspects of DME. Many novel findings are defined subjectively. Further investigation should translate these findings into quantitative parameters to introduce objective diagnosis and assessment. Adaptive optics technologies are now being applied to fundus photography and OCT. Their high resolution delineates cellular changes in the retinas. Based on these fundus findings, translational studies from beds to bench will elucidate the molecular mechanisms in DME. The visible parameters on imaging and the invisible parameters in molecular mechanisms reciprocally elucidate the pathogenesis. Hopefully, these challenges will lead to the development of novel treatments. Furthermore, statistical analyses of prognostic factors are also contributing to the promotion of customized medicine. These integrative advances should improve preventive and therapeutic interventions for DME.

## Figures and Tables

**Table 1:** The correspondence between findings on classical and modern modalities. CFP = color fundus photograph; FA = fluorescein angiography; IA = indocyanine green angiography; OCT = optical coherence tomography; OCTA = optical coherence tomography angiography; SW-FAF = short wavelength fundus autofluorescence; NIR-FAF = near-infrared fundus autofluorescence.

Classical Modalities	Modern Modalities
Diagnosis of diabetic macular edema (DME)	
Clinically significant macular edema on CFP	Center involving DME on OCT
Microaneurysms	
Dot-like reddish lesion on CFP	Oval or round on OCT
Hyperfluorescent dot on FA, IA	Saccular or fusiform appearance on OCTA
Fluorescein leakage on FA	-
Fluorescein pooling on FA	Cystoid macular edema on OCT
Capillary nonperfusion on FA	No boundaries between inner layers on OCTLamellar capillary nonperfusion on OCTA
Choroid	
Hyperfluorescent or hypofluorescent areas on FA, IA	Flow void (or deficit) in the choriocapillaris layer on OCTA
	Reduced vascular density or tortuosity of choroidal vessels on OCT
-	Serous retinal detachment on OCT
-	Disrupted or absent ellipsoid zone line on OCT
Hard exudates on CFP	Hyperreflective foci on OCT
-	Disorganization of the retinal inner layers
-	Epiretinal membrane or vitreomacular traction on OCT
Retinal pigment epitheliopathy on CFP	Reduced autofluorescence on SW-FAF
	Mosaic pattern on NIR-FAF

## Data Availability

Not applicable.
